# Circulating Tumour Cells, Cell Free DNA and Tumour-Educated Platelets as Reliable Prognostic and Management Biomarkers for the Liquid Biopsy in Multiple Myeloma

**DOI:** 10.3390/cancers14174136

**Published:** 2022-08-26

**Authors:** Alessandro Allegra, Gabriella Cancemi, Giuseppe Mirabile, Alessandro Tonacci, Caterina Musolino, Sebastiano Gangemi

**Affiliations:** 1 Division of Hematology, Department of Human Pathology in Adulthood and Childhood “Gaetano Barresi”, University of Messina, 98125 Messina, Italy; 2 Clinical Physiology Institute, National Research Council of Italy (IFC-CNR), 56124 Pisa, Italy; 3 Allergy and Clinical Immunology Unit, Department of Clinical and Experimental Medicine, University of Messina, 98125 Messina, Italy

**Keywords:** multiple myeloma, liquid biopsy, cancer biomarkers, circulating tumour cells, cell free DNA, miRNAs, exosomes, tumour-educated platelet, prognosis

## Abstract

**Simple Summary:**

Even though the presently employed biomarkers in the detection and management of multiple myeloma are demonstrating encouraging results, the mortality percentage of the malignancy is still elevated. Thus, searching for new diagnostic or prognostic markers is pivotal. Liquid biopsy allows the examination of circulating tumour DNA, cell-free DNA, extracellular RNA, and cell free proteins, which are released into the bloodstream due to the breakdown of tumour cells or exosome delivery. Liquid biopsy can now be applied in clinical practice to diagnose, and monitor multiple myeloma, probably allowing a personalized treatment of the disease.

**Abstract:**

Liquid biopsy is one of the fastest emerging fields in cancer evaluation. Circulating tumour cells and tumour-originated DNA in plasma have become the new targets for their possible employ in tumour diagnosis, and liquid biopsy can define tumour burden without invasive procedures. Multiple Myeloma, one of the most frequent hematologic tumors, has been the target of therapeutic progresses in the last few years. Bone marrow aspirate is the traditional tool for diagnosis, prognosis, and genetic evaluation in multiple myeloma patients. However, this painful procedure presents a relevant drawback for regular disease examination as it requires an invasive practice. Moreover, new data demonstrated that a sole bone marrow aspirate is incapable of expressing the multifaceted multiple myeloma genetic heterogeneity. In this review, we report the emerging usefulness of the assessment of circulating tumour cells, cell-free DNA, extracellular RNA, cell-free proteins, extracellular vesicles, and tumour-educated platelets to evaluate the changing mutational profile of multiple myeloma, as early markers of disease, reliable predictors of prognosis, and as useful tools to perform less invasive monitoring in multiple myeloma.

## 1. Introduction

Multiple myeloma (MM) is a malignancy due to the clonal growth of plasma cells (PCs); it is a not curable condition and MM subjects undertake heavy therapies with longer or shorter intervals of remission before a relapse happens, thus making a new therapy to start. With the discovery of new drugs and therapeutical procedures, patients’ survival has increased to 50% (five years) and 29% (ten years) in 2018 with respect to 37% of 5-year-survival between 2005 and 2009 [1,2,3].

Several types of MM have been described, between them micromolecular myeloma and extramedullary multiple myeloma (EMM), which is an aggressive form of MM, identified by the presence of tumoral PCs outer of the bone marrow (BM); it is a multi-focal condition characterized by a huge heterogeneity and correlated with a bad outcome [4]. Both forms appear to be more difficult to diagnose than the classic form of the disease.

The identification of MM is performed through the discovery of clinical, chemical, histological, and radiological signs of disease, which considerably modify patient survival [5,6].

BM aspirates constitute the main method employed to identify the disease, perform a prognosis, and define a genetic classification. However, they stand for a relevant shortcoming for serial MM examining as they require a painful and invasive practice. Furthermore, new results displayed that a sole BM aspirate is incapable of expressing the intricate MM heterogeneity. In fact, beyond aggressive, BM aspirates might not be entirely demonstrative due to irregular BM participation, spatial genomic heterogeneity, or the presence of an EM disease. A recent analysis executing multi-region evaluation of iliac crest confirmed the presence of spatial genomic heterogeneity in more than 75% of MM subjects [7]. Thus, there is mounting attention in alternate techniques to identify the MM disease and to evaluate genetic profile of medullar and EM tumour plasma cells.

Liquid biopsy (LB) is the procedure of investigating neoplastic cells, genetic materials (such as DNA, or MiRNAs), tumour-educated platelets or other components in a liquid milieu instead of traditional tissue biopsy; the material used for the liquid biopsy can be from peripheral blood (PB) or any other organic fluids [8].

Although some well-structured reviews have been written on the possibility of using liquid biopsy in MM, often these reviews have privileged an aspect of myelomatous disease such as diagnosis or prognosis, the possibility of detecting the minimal residual disease or to understand some aspects of the biology of myeloma. Our work sought to summarize the different aspects of the relationships of liquid biopsy with the clinic, biology, diagnosis, prognosis and treatment of MM [9,10,11,12].

**Figure 1:**
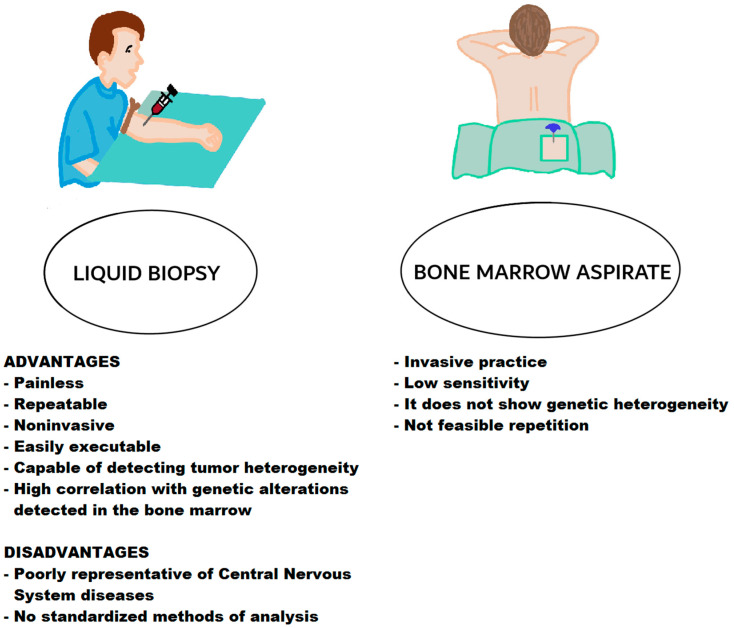
Characteristics of liquid biopsy compared to bone marrow investigations.

In the last few years, several experimentations have confirmed the ability of LB in identifying the disease, considering prognosis, performing a molecular profile, and detecting minimal residual disease (MRD), and have suggested that LB might be employed as a substitution of BM aspirates [13,14] (Figure 1).

## 2. Liquid Biopsy and Circulating Tumour Cells

Movement of circulating tumour cells (CTCs) appears to be an early event in tumorigenesis, and several reports have demonstrated its occurrence in different types of tumours [15,16]. Recently, CTCs have gained importance as they are minimally intrusive markers that can disclose essential data about the neoplastic disease. Actually, CTCs may have several clinical applications, such as in identification, treatment management, and monitoring of cancer patients [17].

In MM, CTCs are discharged from the BM into the blood circulation [18], returning again to the BM at various positions in a spreading course [19]. As CTCs come out straight from the BM, their phenotype should coincide with that of the BM plasma cells [20,21]. In fact, the presence of typical B-cell maturation indicators or other molecules such as adhesion molecules stayed unchanged with respect to BM compartment [22]. Nevertheless, the capacity of CTCs to come out may also imply specific characteristics promoting MM diffusion via systemic circulation, making it a possible sign of different cellular functionality.

### 2.1. Methods of Detection of CTCs

Several new methods to identify CTCs have emerged, either via cytometric techniques or nucleic acid-based procedures [23,24,25,26]. However, cytometric techniques have been demonstrated to be easier to perform and have been preferred to recognize CTCs employing their size, antigen expression, and the presence of specific molecules. For instance, as for solid tumors, CellSearch is an FDA-approved method to identify CTCs of epithelial origin [27,28,29,30]. In spite of the small number of CTCs and morphologic heterogeneity, this method is capable of guaranteeing a correct and reliable evaluation of CTCs [31].

A specific technique performed employing an optical apparatus named “diffuse in vivo flow cytometry” (DiFC) was used to analyse uncommon, green fluorescent protein expressing CTCs [32], and to evaluate short-term modifications of CTCs in MM. For rare CTCs (less than 1 CTC per ml of blood), the use of short time DiFC periods often occasioned no identifications. Instead, for more diffuse CTCs, the number differed by an order of magnitude over the timescales considered. The results were coherent with important modifications in the number of CTCs on short times (minutes and hours). This may be due to the constant detachment of CTCs from BM and the brief half-life of CTCs in the blood. However, authors demonstrated that the evaluation can be improved by analysing greater volumes and numerous samples.

In a different study, researchers used an enrichment-free four-plex immunofluorescence technique and single-cell DNA sequencing for identification and genomic classification of plasma cells to distinguish normal and malignant plasma cells in blood and BM aspirates from patients with newly diagnosed myeloma and monoclonal gammopathy of undetermined significance (MGUS) [33]. The results confirmed that peripheral blood plasma cells presented the same genomic modifications that were present in BM plasma cells. However, a group of rare cells presented genetic changes not identified by conventional diagnostic techniques of random localized BM biopsies. Thus, this type of LB can identify rare cells that have the capacity to improve our knowledge of subclonality in MM.

A different approach was performed by Foulk et al., who developed a different method to analyse CTCs employing fluorescence in situ hybridization (FISH) together with next-generation sequencing (NGS), recognizing a greater number of CTCs in all phases of MM with respect to normal subjects. Furthermore, they were able to find a correlation between CTCs and the number of PCs in the BM, the amount of monoclonal component, and the stage (ISS) of disease [34]. Similarly, a different experimentation allowed for implementing a cell-based immunofluorescence test to differentiate MM CTCs from normal cells based on morphologic characteristics and the presence of antigens, such as CD138 and CD45 [35].

A different method to detect CTCs and BM clonal cells employed the next-generation flow (NGF) cytometry [36]. In some cases, plasma cells from EM plasmacytomas were also evaluated and whole-exome sequencing was made in three spatially distributed samples. CTCs were identified in the PB of all MM subjects. Authors highlighted that about 22% of CTCs came out from a BM or EM place far from the corresponding BM aspirate. All high-risk genetic alterations were identified in CTCs if present in BM plasma cells, excluding one t(4;14). In any case, about 82% of genetic alteration occurring in BM and EM neoplastic cells were demonstrable in CTCs. These data support the ability of CTCs as useful, non-invasive risk-stratification tool of MM patients.

Recently, Wang et al. employed a mixed aptamer-triggered hybridization chain reaction with tetrahedral DNA framework, herringbone channel chip, to create an effective microfluidic system for analysis of simulated CTCs [37], while a new method for in-situ separating and rapidly identifying CTCs from peripheral blood at single-cell resolution employing black TiO_2_-based Surface-Enhanced Raman Scattering bio-probe on a microfilter was also proposed [38]. CTCs were extracted from peripheral blood by microfilter according to the size and deformation difference. The bio-probe was constituted of crystal-amorphous core-shell B-TiO_2_ nanoparticles, alizarin red as Raman reporter molecules, and a fine protecting protective stratum of NH_2_-PEG2000-COOH.

Other studies confirmed that CTC mutations at a single-cell level agreed with BM plasma cells [39]. However, different results are present in literature, and in a study only 20% genetic alterations were present in both neoplastic compartments. Conversely, employing a wider approach as the whole exome sequencing (WES), CTCs were able to present up to 93% of genetic alterations identified in BM plasma cells [40] (Table 1).

Transcriptomic analysis has been commonly employed to classify MM subgroups with specific characteristics and different outcomes [41]. Overall, the extremely coinciding transcriptomic findings suggest that CTCs mirror their BM equivalent. Thus, models tend to cluster in a patient-specific modality rather than by MM cell source [42,43]. This hypothesis would be further confirmed by the relevant similarity between CTCs and BM plasma cells upon reconstructing the B-cell receptor (BCR), which indicates the identical clonal derivation.

From what has been said, it is evident that we are far from the possibility of standardizing the CTC evaluation technique. Each of the proposed methods seems to have advantages and offers the possibility of detecting characteristics of the biology of MM. Thus, the differential expression of several molecules such as adhesion molecules, integrins and cytokine receptors might be useful to clarify specific dynamics of specific MM subsets [44]. For instance, the decreased presence of CD81 and CD138 supports the possibility that, in some cases, CTCs could be a more immature population. Instead, the absence of CD49 and CD56 has been correlated with an aggressive disease and suggested that it was a probable sign of plasma cell leukemia [45,46].

Probably, in the future, it will be necessary to privilege the reproducibility and comparison of the results obtained using techniques whose validity is widely shared.

### 2.2. CTC, Progression and Prognosis of Monoclonal Gammopathies

It is well known that MGUS evolves at a uniform and small percentage of 1%/year. The chance of converting from smouldering multiple myeloma (SMM) to active MM is more varied. Various risk elements have been recognized to calculate SMM risk advancement at diagnosis, and scales have been suggested to detect subjects with an increased risk of progression at two years. It is possible that employing different scales some subjects might be incorrectly classified as SMM while really holding MM. This emphasizes the requirement for improved diagnostic criteria to recognize higher risk SMM subjects that may profit from precocious therapy [47].

CTCs can be found almost immediately after the premalignant clonal process has started. A recent study displayed that 59% of subjects affected by MGUS have demonstrable CTCs with respect to 100% of cases with SMM and manifest MM [48]. The main distinction between these conditions is the rate of CTCs, with increasing percentages from 0.0002% (0.008 CTCs/µL—for MGUS) to 0.004% (0.16 CTCs/µL—for SMM) and 0.04% (1.9 CTCs/µL—for overt MM) [49]. Significantly, the number of CTC was considered an independent prognostic element with respect to BM morphology or FC. Increasing logarithmic ratios of CTCs were correlated with lower progression-free survival. A cut-off of 0.01% CTCs displayed an independent prognostic significance (hazard ratio: 2.02) in multivariate progression-free survival analysis including lactate dehydrogenase concentrations, the International Staging System, and cytogenetic analysis. MM subjects with undetectable CTCs had extraordinary progression-free survival regardless of complete remission and MRD condition. In patients with detectable CTCs, only obtaining MRD negativity displayed a significant increase in progression-free survival [50].

However, an experimentation employed single cell RNA sequencing to analyse subjects along the monoclonal gammopathy advancement spectrum, revealing great interindividual variation. In any case, in subjects with initial disease and in patients who presented a minimal residual disease after therapy, authors identified only few clonal PCs with molecular findings analogous to those of patients with advanced myeloma. Thus, this method allows the employ of LB to detect tumoral PCs, which reproduce BM disease [42].

Furthermore, genomic analysis of CTCs through whole exome sequencing has confirmed the existence of a great correspondence in clonal alterations between CTCs and BM samples in the different phases of diseases, although some sub-clonal alterations were identified only in CTCs [51,52,53].

Liquid biopsy has also been used in the set of MM patients undergoing transplantation. In a phase III clinical trial, subjects with MM were analysed at the diagnosis, after treatment (achievement of complete response, after induction treatment, autologous stem cell transplant (ASCT)) and consolidation treatment [53]. A 99.6% decrease in the number of CTCs was reported from diagnosis to post-ASCT. Remarkably, ISS III MM subjects only displayed a decrease in CTCs after ASCT, and not after that the induction treatment has been performed alone.

### 2.3. CTCs and Prognosis of Multiple Myeloma

Many reports have confirmed the presence of a correlation between the number of CTCs and parameters correlated with a poor prognosis [20,54]. The quantity of CTCs was reported to be a marker of survival in subjects with newly identified and relapsed MM [55]. When the evaluation was limited to the actively relapsing patients, the best cut-off predicting for the greatest risk of death within a year was about 100 clonal CTCs; with a sensitivity of 62% and specificity of 73%. According to this result, authors identified ≥100 events as a cut-off for labelling the prognostic value of CTCs in actively relapsing MM subjects [56]. Remarkably, a high number of CTCs in SMM were correlated with a huge risk of progress to manifest MM in 2–3 years after diagnosis [57]. Lately, employing the NGF technique Sanoja-Flores et al. confirmed that CTCs in PB at the disease onset are linked to a bad prognosis of both MGUS and MM subjects [58].

Experimental studies have suggested that CTC clusters might have a prognostic significance and might be able to predict chemoresistance. These clusters can provide data on modifications in the genetic outlines, somatic changes, and epigenetic alterations with respect to cells in a primary tumour site. Microfluidic techniques have the possibility of investigating CTC clusters via the capability to proficiently separate these cells from the peripheral blood of patients in a liquid biopsy. Microfluidics can also be employed in in vitro experimental models of CTC clusters and allow their identification and study [59,60,61,62].

## 3. Liquid Biopsy and Cell Free DNA

### 3.1. Origin of Cell Free DNA

The greater part of cell free DNA (cfDNA) in PB derives from hematopoietic cells [63] and tumour-originated cfDNA (ctDNA) can be present in very small amounts, according to the stage of disease and the form of tumour [64]. However, cfDNA in MM has been reported to have a great similarity with BM aspirates, although some studies have displayed that this concordance is generally limited to a restricted number of mutations [65,66]. This could be judged a shortcoming due to the great inter- and intra-patient genetic heterogeneousness of MM [67], and the consideration that copy number alterations are the keystone of disease prognosis [68,69]. However, as reported above, WES of cfDNA offers more data on genetic characteristics and has also demonstrated greater similarity with BM aspirates [70].

Nucleic acids are discharged into the peripheral blood after cellular death due to programmed cell death, or necrosis, and the spontaneous liberation of DNA/RNA-lipoprotein complexes [71].

The possibility to find cf nucleic acids in the serum was first reported decades ago [72]. Several studies performed on solid tumours demonstrated that neoplastic cells could liberate nucleic acids and that this cfDNA is representative of the whole genome of the tumour. Compared to normal subjects (mean: 15.21 ng/mL; median: 14.37 ng/mL; range: 12.20–19.51 ng/mL), tumour subjects always displayed greater concentrations of plasma DNA (mean: 275.35 ng/mL; median: 139.0 ng/mL; range: 22.44–1037.49 ng/mL) (*p* < 0.0001) [73]. Furthermore, genome sequencing and WES of the cfDNA might be employed to recognize genetic alterations correlated with chemoresistance without the need to perform successive biopsies. A study recognized a subset of genes that were positively selected after therapy, many of which have been correlated before with drug resistance (MED1, GAS6, PIK3CA) [74].

The size of cfDNA ranges from 50 base pairs to 1000 base pairs, but generally ranges between 50 and 200 base pairs [75]. It has a short life (4–30 min), as it is quickly eliminated by the hepatic and renal system and circulating DNase [76]. Normal subjects generally present small amounts of cfDNA, but concentrations are much increased in tumours. Nevertheless, any situation able to cause a foster destruction or death of non-tumoral cells will also enhance cfDNA concentrations. These conditions include inflammation, infections, and trauma [77,78].

It is noteworthy that serum contains 20 times more cfDNA than plasma as it is discharged from the white cells when they are damaged. However, this may reduce the cfDNA deriving from the tumour. Thus, plasma is the favoured material employed to decrease background interference during the test [79].

### 3.2. Methods of Detection of cfDNA

The use of the deep sequencing of ctDNA from MM subjects is an extremely sensitive method able to recapitulate mutational patterns of corresponding BM aspirates [80]. Droplet digital PCR is presently employed to analyse mutational status and to track MM progression [81]. Kis et al. displayed that analysing of ctDNA allows the study of sub-clonal hierarchies, mirroring BM samples [65]. Nevertheless, in some cases, the genetic alterations recognized were identified only in plasma, which is coherent with the spatial heterogeneity of MM [82,83,84]. In this sense, integrating plasma ctDNA assessment in MM evaluation might constitute a relevant progress in the effort to individualize MM treatment approaches [85].

It was proposed that the minor tumour load of early phases possibly relates with a smaller cfDNA amount. Even with the more effective techniques, more than 50% of asymptomatic subjects did not display any alteration in cfDNA, probably indicating the low mutational bulk of these conditions [86]. These data support the hypothesis that ichor copy number alteration (CNA) execution is inadequate in the group of patients with small tumour elements. However, since ichor CNA, an algorithm optimized for ultralow pass sequencing, assesses the rate of tumour-originated cfDNA via the identification of somatic CNAs, it is conceivable that, in subjects lacking clonal CNAs, the tumour portion could be undervalued. To improve the employ of LB, a more adequate genome-wide strategy could be applied. Nevertheless, the cost–benefit proportion should be evaluated at the economic side. 

Kis et al. described a hybrid-capture-based LB Sequencing (LB-Seq) technique employed to evaluate all protein-coding exons of BRAF, EGFR, KRAS, NRAS, and PIK3CA in cfDNA samples from MM patients. This methodology includes a filtering algorithm that allows identification of tumour-originated portions existing in cfDNA at allele incidences as low as 0.25%. Employing this method, they identified 49/51 somatic mutations, with subclonal hierarchies reproducing tumour profiling with a concordance of 96%, and other further mutations probably skipped to BM aspirates [65]. These findings were confirmed by other studies [66].

Between the advantages of cfDNA, there is the opportunity with which it can be managed. With common accessible phlebotomy tubes that enclose formaldehyde-free fixatives, cfDNA is constant at room temperature for several days [87]. However, plasma should be achieved by centrifugation within hours after sample attainment to prevent cell death and DNA alteration, as DNA from white blood cell destruction may reduce the levels of MM-originated cfDNA [88].

Nevertheless, one of the inconveniencies of cfDNA is that it cannot be enhanced for MM-specific cfDNA, dissimilar MM cells from the BM, which can be enhanced employing magnetic bead sort or flow selection. In any case, it is much easier to increase the amount of cfDNA by enhancing the quantity of PB extracted than augmenting the quantity of BM obtained through a biopsy.

### 3.3. cfDNA Disease Advancement and Relapse

Amounts of cfDNA seems to connect to spread and severity of the disease. To evaluate the correlation between cfDNA and MM progression, a study considered plasma samples from MM subjects without extramedullary involvement and EMM patients. Remarkably, greater amounts of cfDNA were achieved from patients who presented extramedullary involvement with respect to subjects without this type of involvement. Furthermore, plasma ctDNA presented a greater concordance to extramedullary tumour than that of BM aspirates. Thus, cfDNA is a useful substitute for EMM genetic determination, especially when tumour is difficult to get to, and may also be employed to trace MM progress [89].

As said above, SMM is a predecessor condition of MM, in which some subjects quickly evolve to MM, while others maintain this dormant type of the disease [90]. Many studies have investigated genomic characteristics in SMM that can correctly identify patients with high risk SMM [91,92]. However, there are only limited studies on cfDNA in SMM. From some reports, cfDNA was reduced in SMM subjects with respect to patients with overt MM. An experimentation evaluated if cfDNA amount changes corresponding to risk level classified by the 70 gene expression profile (GEP70) [93]. cfDNA concentrations were remarkably greater in the GEP70 high risk set with respect to the low risk set and were associated, albeit faintly, with different biomarkers such as β2-microglobulin, and lactate dehydrogenase. Furthermore, authors evaluated cfDNA amounts in SMM subjects performing serial analyses and displayed increased allele portion of mutated KRAS as cfDNA increases. These findings state that cfDNA is a dynamic instrument able to catch genetic changes in monoclonal gammopathies [93].

In a different study, the usefulness of cfDNA to identify the tumour load and to predict the possibility of a disease relapse in MM subjects was proved. Furthermore, the relapse of MM was evidenced more accurately by evaluating cfDNA with driver mutations than by evaluating serum free light chains [94].

The prognostic ability of cfDNA was also demonstrated in a different setting of patients such as novel diagnosed (ND) and relapse/refractory (RR) subjects, studying ras/raf signaling pathway and TP53 in BM and plasma samples. The whole amount and percentages of genetic alterations in the different type of samples were remarkably greater in RR subjects with respect to ND subjects. Furthermore, subjects featuring more than two mutations presented a remarkably shorter overall survival (OS). Similarly, subjects with TP53 alterations had shorter OS with respect to subjects with no TP53 alterations. Finally, cfDNA evaluation recognized a greater occurrence of DNA-repair gene altered subclones than BM examination [94].

Rustad et al. reported a connection between the number of mutated alleles in the PB and the proportion of BM plasma cells, which may mirror mutated cells, the total tumour load, and the progression to a more severe condition that is corresponding to the M protein [81].

Vravel et al. published a long-term analysis on blood-based MRD assessment employing tumour-specific cfDNA identification by ASO-qPCR [95]. They highlighted a relevant connection of amount of tumour-specific cfDNA concentrations with clinically important situations such as induction treatment and autologous stem cell transplant (ASCT). Moreover, size of cfDNA fragments is correlated with improved therapy response.

All these data sustain the idea of tumour-specific cfDNA as a marker of prognosis, since a greater amount of plasma genetic alterations is correlated with a reduced survival [95]. This notion is coherent with the assumption that increased spatial genetic heterogeneity constitutes an unfavorable condition in MM patients, with a higher probability of chemoresistance. Thus, detecting such heterogeneity may be essential to identify patients who might be unresponsive to treatment. In fact, resistance to a specific substance is likely due to the occurrence of driver genetic alterations in oncogenes or tumour suppressor genes. Surely, these alterations are more recognizable with LB than with a single-site BM aspirate. Evaluating the genetic profile through liquid biopsies would offer a more illuminating opportunity for personalised treatment in MM [96].

Remarkably, certain mutations of great prognostic importance such as PIK3CA are more easily identified by a LB [65,66], and cfDNA may aid in recognizing MM subjects with an adverse risk profile more adequately than only via BM aspirate [97].

### 3.4. cfDNA and MRD Evaluation

Regarding the study of the MRD with cfDNA, there are some reports that compare it to the other conventional techniques such as flow cytometry and DNA evaluation in BM [98]. Mazzotti et al. displayed the lack of a connection between DNA and BM for the MRD by NGS, employing only IgH gene rearrangements [99]. A different study demonstrated that cfDNA can be employed to evaluate the MRD, especially in EMM [89]. However, in other experimentations, although a relevant correlation between the amount of tumour-specific cfDNA with clinical events has been identified, the data in the case of the MRD checking were not meaningful [51].

Currently, the application of cfDNA alone has no employ in the evaluation of the MRD in MM patients, although there is increasing evidence that it might be a useful ancillary procedure for the management and control of the pathology and, perhaps, with its perfection, into a relevant instrument for the assessment of the MRD [100].

Finally, subjects with non-secretory MM might also profit from cfDNA evaluation as monoclonal proteins or serum free light chains are not obtainable as biomarkers for checking these subjects [101,102]. Furthermore, cfDNA assessment may also be used for fragile and elderly MM subjects with different, relevant conditions, which are less subjected to repeated BM aspirates. For these subjects, cfDNA analysis is markedly appealing, as this procedure allows indolent evaluation, which can be performed by sending the samples to laboratories far away and allows complete molecular investigation without the need for invasive practices (Table 2).

## 4. Liquid Biopsy and MiRNAs

### Methods of Detection and Biological Significance of miRNAs

MicroRNAs (miRNAs) are a group of small molecules of 19–24 nucleotides in size, and they are the most copious RNAs in the peripheral blood. They have a great stability and perform a relevant action in MM proliferation and in the onset of chemoresistance [103,104,105,106].

Until now, several established laboratory procedures have been employed for the evaluation of miRNAs, including oligonucleotide microarray, qRT-PCR, and northern blotting [107]. Recently, an integrated miRNA biosensing method for the detection and analysis of serum-based miRNAs employing the DNA-linked gold nanoprobe and duplex-specific nucleases-mediated signal amplification was proposed [108].

Chen et al. assessed the possibility to use extracellular RNA (exRNA) originated from the peripheral blood of MM subjects for entire transcriptome definition [109]. They recognized 632 differentially-expressed genes (DEGs) in MM subjects, of which 26 were shared in NDMM and RRMM patients. Furthermore, they recognized 54 and 191 genes exclusive to NDMM and RRMM patients, and these included several miRNAs [109].

In a study, quantum dot-molecular beacon functionalized MoS2 fluorescent probes were employed for the simultaneous identification of MM-correlated miRNA-155 and miRNA-150 [110]. The two probes can efficiently find miRNA-155 and miRNA-150 with adequate recovery percentages, with a low detection limit. These findings suggest that this technique is the most precise method to analyse miRNAs and that fluorescent probes with signal amplification approaches can obtain extremely sensitive identification of MM-correlated miRNAs in MM patients.

## 5. Liquid Biopsy and Exosomes

### 5.1. Origin and Detection of Exosomes

A different form of LB consists of the evaluation of the genetic material enclosed in the exosomes discharged by the neoplastic cells. In fact, some experimentations have revealed the presence of miRNAs and long noncoding RNAs in the exosomes extracted from the peripheral blood of MM subjects [111,112,113].

Small extracellular vesicles (EVs) or exosomes are elements of about 30–150 nm in diameter surrounded by a lipid bilayer, which are delivered from cells into the extracellular milieu and perform relevant actions in several biological and pathological phenomena including tumours [114,115].

### 5.2. Biological and Clinical Significance of Exosomes

EVs are capable of regulating the activities of target cells by modulating metabolic and signalling paths, and in a tumoral setting, promoting the generation of an advantageous tumour micromilieu [116,117]. EVs present similar biomarkers of their cell of origin, and this allows for evaluating them as a useful target for identification and treatment of cancer patients [118]. Relevantly, EVs defend their cargo including RNAs from destruction in the extracellular space and can be efficaciously isolated from peripheral blood, making them perfect elements for LB [119,120].

To date, different analytical techniques have been implemented for the exosome identification. For instance, exosomes can be isolated and studied by ultra-centrifugation, and isolation kits are commercially available. Furthermore, the features of exosomes can be quickly studied by enzyme-linked immunosorbent assay and Western blot analysis or employing the scanning or transmission electron microscopy, nanoparticle tracking examination and atomic force microscopy. The identification techniques also include the polymerase chain reaction or sequencing techniques. Moreover, several new sensing methods have been utilized in the exosomes study, such as colorimetric detection, fluorescence assay, surface plasmon resonance, electrochemical analysis, and integrative microfluidic systems. Recently, the electrochemical analysis with the nano-sensing interface has been proposed [121,122,123,124].

Exosome gene components seem to conserve the same abilities as far as diagnoses and prognosis as cfDNA. For instance, many experimentations have evaluated the capability of EV-RNA to discriminate between MGUS or MM subjects at the disease onset, together with and other hematological pathologies. Caivano et al. reported the diagnostic capability for small EV-originated miRNA-155. Peripheral blood concentrations were displayed to be remarkably reduced in MM subjects with respect to other different hematologic pathologies [125]. Analogously, EVs originated from the peripheral blood of MGUS, or MM subjects presented considerably lower concentrations of lncRNA PRINS with respect to different hematological diseases [113]. Kubiczkova et al. stated that miRNA-34a and let-7e that originated from circulating exosomes can differentiate MGUS and MM from other diseases with extreme accuracy [126]. Similarly, Zhang et al. [127] displayed that peripheral blood-originated miRNA-20a-5p, miRNA-103a-3p, miRNA-185-5p, miRNA-425-5p, let-7c-5p, and let-7d-5p concentrations were extremely reduced in exosomes extracted from MM subjects with respect to different hematological diseases, while the concentrations of miRNA-4505 and miRNA-4741 were considerably greater. As for monoclonal gammopathies, lower concentrations of exosome-originated miRNA-20a-5p, miRNA-103a-3p and increased concentrations of miRNA-425-5p, and miRNA- 4505 were also capable of differentiating SMM from different hematological diseases. Furthermore, authors also demonstrated that reduced concentrations of exosome-derived miRNA-20a-5p, miRNA-103a-3p, miRNA-140-3p, miRNA-185-5p, let-7c-pc and greater concentrations of miRNA-4505 and miRNA-4741 were able to discriminate MM with respect to SMM patients. The study of the genetic material derived from exosomes could also provide useful indications regarding the tendency to the disease progression. Exosomes extracted from the peripheral blood of MM subjects were reported to present altered concentrations of let-7b and miRNA-18a, which are involved in MM progression [112]. Lower levels of both let-7b and miR-18a were significantly associated with poor prognosis with a reduced progression-free survival and OS in NDMM subjects treated with bortezomib-based treatment, validating their prognostic significance and their usefulness for risk definition of chemoresistance [112].

The latter is an extremely relevant argument in MM patients. Multidrug resistance (MDR) is a specific form of chemoresistance, in which MM cells develop cross-resistance to a broad variety of different, unrelated substances after contact with a single drug [128,129,130].

A study recognized large EVs that can be employed to evaluate clonal load, MM diffusion and the onset of MDR in MM patients [131]. The cargo enclosed in EVs differed in the presence of several biomarkers such as CD138, CD34, P-glycoprotein (P-gp), and phosphatidylserine (PS). Augmented concentrations of P-gp and PS correlated with MM advancement and failure to respond to treatment. Moreover, PS, P-gp and CD34 were essentially present in CD138− EVs in progressed MM. Specifically, a population positive for CD138-P-gp+CD34 is augmented in advanced and unresponsive MM [131]. This analysis constitutes a test performed via LB able to manage MDR and non-responsiveness to treatment (Table 3).

In conclusion, Evs constitutes an adequate substitute of their cells of origin, which are mostly restricted to the BM. MDR in MM subjects MM can be recognized and successively controlled by evaluating exosomes in peripheral blood in the field of liquid biopsies.

## 6. Tumor-Educated Platelets (TEP) and Liquid Biopsy

Platelets are circulating corpuscles deriving from megakaryocytes in the BM, which have a main role in haemostasis and the beginning of wound healing. However, they also participate in general and local phenomena linked to tumour proliferation, as neoplastic cells can modify the RNA pattern of these elements. Furthermore, TEPs can absorb the circulating mRNA delivered by neoplastic cells or ingest solubilized tumour-derived proteins [132]. These connections between platelets and tumour cells may imply a possible role of TEPs for tumour identification and management [133].

**Figure 2:**
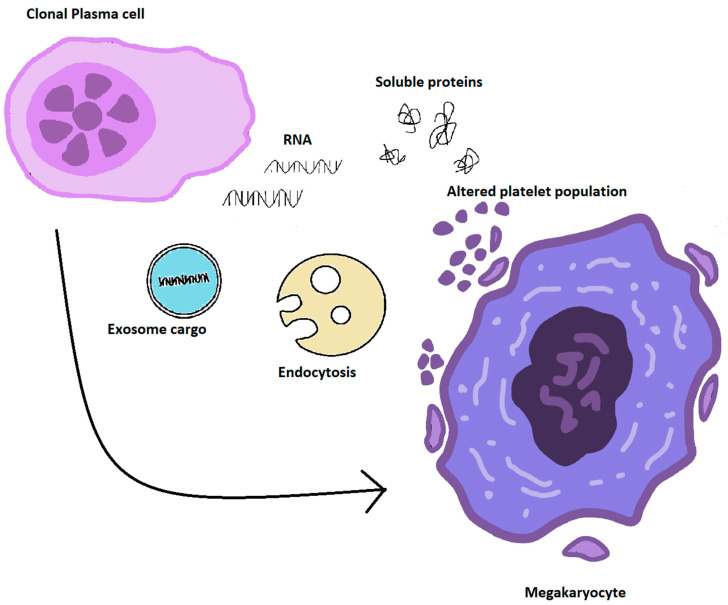
Tumour educated platelet in liquid biopsy.

Neoplastic cells can relocate molecules such as RNA to platelets via direct connection and delivery of exosomes, which is able to modify the platelet precursor RNA, and under the effect of neoplastic cells and neoplastic milieu platelet immature mRNA is transformed into mature RNA able to form functional proteins, generating TEPs (Figure 2). The identification of TEPs in the peripheral blood of neoplastic subjects is awaiting employment in the tumour diagnosis [134,135,136] (Table 3).

**Table 3:** Studies on exosomes and educated platelets.

	Methods	Targets	Results	Refs.
Prognostic significance	Small RNA sequencing analysis and quantitative reverse transcription polymerase chain reaction array	Circulating exosomal microRNAs	miRNAs, let-7b and miR-18a, were significantly associated with PFS and OS	[112]
	TaqMan Low Density miRNA Arrays	Expression of 667 miRNAs	Lower levels of miR-744 and let-7e were associated with shorter overall survival and remission of myeloma patients	[126]
	Flow cytometric analysis	Analysis of circulating large extracellular vesicles	Elevated levels of P-glycoprotein and phosphatidylserine correlate with disease progression and treatment unresponsiveness	[131]
Diagnostic significance	RT2 lncRNA PCR Array—Human lncRNA Finder	Long noncoding RNA molecules	Dysregulation of exosomal lncRNA PRINS in MM vs. controls	[113]
	Quantitative RT-PCR	Exosome miR155 levels	Exosome miR155 levels were significantly lower in multiple myeloma vs. controls	[125]
	Real-time quantitative PCR	Expression of let-7c-5p, let-7d-5p, miR-140-3p, miR-185-5p, and miR-425-5p	Expression of exosomal miRNA is related to the expression levels of a clinical feature-related factor	[127]
Multiple myeloma educated platelets				
Prognostic significance	Hematological analyzer	Evaluation of mean platelet volume in patients with multiple myeloma	Low mean platelet volume is correlated with poor prognosis in MM patients	[135,136,137]

Although there are no specific studies in progress about TEPs and MM, several correlations between platelets and MM have been identified, such as the notion that low mean platelet volume is correlated with worse outcome in MM subjects and may be employed as a relevant marker for MM advancement and prognosis of these patients [137,138]. Soon, the analysis of TEPs could constitute a useful complement in the context of liquid biopsies and a new approach to myeloma disease.

## 7. Conclusions

Latest developments in genomic knowledge have not only enhanced our understanding of the molecular mechanisms of hematological diseases, but also modified the area of clinical diagnostics.

In this field, the employ of liquid biopsies seems a promising tool to be used also in MM subjects. Until now, experimentations focused on CD138+ augmented CTCs or on cfDNA for genetic analysis in MM patients have confirmed their usefulness for diagnosis, prognosis, and response evaluation [139].

In the future, several features of liquid biopsies will be studied more in-depth to improve results and support their clinical employ. An interesting aspect could be to investigate diverse biomarkers at the same time to obtain higher sensitivity and specificity. For instance, liquid biopsies simultaneously evaluating CTCs and cfDNA might be particularly advantageous. Understanding the genomic concordance of CTCs and cfDNA may modify their employ in performing MM precision medicine.

Furthermore, a fundamental application of liquid biopsies could be correlated to the identification and molecular characterization of EMM. Recent imaging procedures suggest that EMM could be more common than supposed before, and it has been proposed that liquid biopsies might be useful to diagnose and manage EMM [140,141].

Liquid biopsies may be taken into account as a good clinical possibility thanks to their non-invasiveness, absence of painpainless, fair accuracy and reliability. However, before being used to the clinical setting, this technique needs to be confirmed and supported in adequate clinical trials. One of the essential requisites is the integration of these procedures in the managing strategy of MM patients and the achievement of clear evidence of full agreement with the conventional gold-standard parameters including M protein level, BM aspirate, and imaging. The employ of LB in assessing the response to treatment of MM constitutes a further possibility, and in a short time this method could even establish itself as a reference technique in the study of patients with MM.

Surely, there are still several weaknesses to overcome, such as enhancing the achievement of an appropriate amount of cfDNA and the choice of the most appropriate technique to be used (for instance NGS).

The addition of LB into controlled clinical trials will assess its significance as an independent factor for diagnosis and prognosis of MM patients beyond presently existing biomarkers. The practicality and facility of a simple blood draw over a BM aspirate is of great advantage for MM patients and intensely justifies its further exploration in the context of MM.

## Figures and Tables

**Table 1:** Different techniques used in the identification of CTCs.

Technique	Target	Marker	Features	Refs.
DiFc	To evaluate short time modifications	Green fluorescent protein-expressing CTCs	Small samples are unlikely to yield a quantitatively accurate estimate of mean CTC numbers.	[32]
Immunofluorescence technique and single cell DNA sequencing	Identification and genomic classification	CD138, CD56, CD45, DAPI	Detection of rare cell populations	[33]
FISH and NGS	Correlation between CTCs and clinical parameters	CD38, CD38/138, CD45, CD19	It can be used to study disease biology and monitor clinical disease progression	[34]
Immunofluorescence test	To differentiate normal and clonal cells	CD138, CD45, phospho-ribosomal protein S6	The assay is highly repeatable and reproducible	[35]
NGF cytometry	Evaluation of EMM	Patient-specific aberrant phenotypes identified with NGF were used for highly purified fluorescence-activated cell sorting of CTCs	Concordance between BM tumor cells and CTCs was high for chromosome arm-level copy number alterations (≥95%) though not for translocations (39%).	[36]
WES	Correlation with genetic alterations found in BM samples	CD138	100% of clonal mutations in patient BM were detected in CTCs and 99% of clonal mutations in CTCs were present in BM MM.	[40]

DiFc: Diffuse in vivo flow cytometry; FISH: Fluorescence in situ hybridization; NGS: Next generation sequencing; NGF: Next generation flow cytometry; WES: Whole exome sequencing.

**Table 2:** Diagnostic and prognostic value of cfDNA in multiple myeloma.

Diagnosis	Techniques	Target	Features	Refs.
**Extramedullary MM**	CAPP-seq ultra-deep targeted next-generation sequencing	Identification of cancer-gene somatic mutations	Greater amounts of cfDNA with respect to subjects without EMMM	[80]
**Multiple myeloma**	Digital droplet polymerase chain reaction analysis	Evaluation of recurrent mutations, mainly in mitogen activated protein kinase pathway genes NRAS, KRAS and BRAF	Correlation between the number of mutated alleles in the PB and the proportion of BM plasma cells	[81]
**Smoldering MM**	Gene expression profile	Analysis of 70 gene expression	Reduced amounts of cfDNA with respect to overt MM	[93]
**Prognosis**				
**Relapsed MM**	Next-generation sequencing	Identify driver mutations	Percentage of ras/raf and TP53 mutations was remarkably greater in RR subjects with a reduced OS	[94]
**MRD assessment**				
**Extramedullary MM**	Targeted deep sequencing and ddPCR assays	Mutational analysis	The concordance of plasma ctDNA to extramedullary tumor concordance was 0873, which is much higher than that of BM aspirates	[89]
**Multiple myeloma**	Ultra-low-pass whole genome sequencing	Detectability of cfDNA and CTCs	Correlation with disease progression is higher for CTCs	[51]

## References

[1] Statistics of N (2019). Cancer Survival by Stage at Diagnosis for England. https://www.ons.gov.uk/peoplepopulationandcommunity/healthandsocialcare/conditionsanddiseases/datasets/cancersurvivalratescancersurvivalinenglandadultsdiagnosed.

[2] Davies F.E., Pawlyn C., Usmani S.Z., San-Miguel J.F., Einsele H., Boyle E.M., Corre J., Auclair D., Cho H.J., Lonial S. (2022). Perspectives on the Risk-Stratified Treatment of Multiple Myeloma. Blood Cancer Discov..

[3] Allegra A., Innao V., Gerace D., Vaddinelli D., Musolino C. (2016). Adoptive immunotherapy for hematological malignancies: Current status and new insights in chimeric antigen receptor T cells. Blood Cells Mol. Dis..

[4] Touzeau C., Moreau P. (2015). How I treat extramedullary myeloma. Blood.

[5] João C., Coelho I., Costa C., Esteves S., Lucio P. (2015). Efficacy and safety of lenalidomide in relapse/refractory multiple myeloma--real life experience of a tertiary cancer center. Ann. Hematol..

[6] Kumar S.K., Dispenzieri A., Lacy M.Q., Gertz M.A., Buadi F.K., Pandey S., Kapoor P., Dingli D., Hayman S.R., Leung N. (2014). Continued improvement in survival in multiple myeloma: Changes in early mortality and outcomes in older patients. Leukemia.

[7] Rasche L., Chavan S.S., Stephens O.W., Patel P.H., Tytarenko R., Ashby C., Bauer M., Stein C., Deshpande S., Wardell C. (2017). Spatial genomic heterogeneity in multiple myeloma revealed by multi-region sequencing. Nat. Commun..

[8] Ponti G., Manfredini M., Tomasi A. (2019). Non-blood sources of cell-free DNA for cancer molecular profiling in clinical pathology and oncology. Crit. Rev. Oncol. Hematol..

[9] Colmenares R., Álvarez N., Barrio S., Martínez-López J., Ayala R. (2022). The Minimal Residual Disease Using Liquid Biopsies in Hematological Malignancies. Cancers.

[10] Charalampous C., Kourelis T. (2022). Minimal Residual Disease Assessment in Multiple Myeloma Patients: Minimal Disease With Maximal Implications. Front. Oncol..

[11] Wallington-Beddoe C.T., Mynott R.L. (2021). Prognostic and predictive biomarker developments in multiple myeloma. J. Hematol. Oncol..

[12] Soliman A.M., Das S., Teoh S.L. (2021). Next-Generation Biomarkers in Multiple Myeloma: Understanding the Molecular Basis for Potential Use in Diagnosis and Prognosis. Int. J. Mol. Sci..

[13] Oberle A., Brandt A., Voigtlaender M., Thiele B., Radloff J., Schulenkorf A., Alawi M., Akyuz N., Marz M., Ford C.T. (2017). Monitoring multiple myeloma by next-generation sequencing of V(D)J rearrangements from circulating myeloma cells and cell-free myeloma DNA. Haematologica.

[14] Levin A., Hari P., Dhakal B. (2018). Novel biomarkers in multiple myeloma. Transl. Res..

[15] Sundling K.E., Lowe A.C. (2019). Circulating tumor cells: Overview and opportunities in cytology. Adv. Anat. Pathol..

[16] Krebs M.G., Metcalf R.L., Carter L., Brady G., Blackhall F.H., Dive C. (2014). Molecular analysis of circulating tumour cells–biology and biomarkers. Nat. Rev. Clin. Oncol..

[17] Potaro P., Lotey N. (2015). Role of circulating tumor cells in future diagnosis and therapy of cancer. J. Cancer Metastasis Treat.

[18] Ghobrial I.M. (2012). Myeloma as a model for the process of metastasis: Implications for therapy. Blood.

[19] Krebs M.G., Hou J.-M., Ward T.H., Blackhall F.H., Dive C. (2010). Circulating tumour cells: Their utility in cancer management and predicting outcomes. Ther. Adv. Med. Oncol..

[20] Paiva B., Paino T., Sayagues J.-M., Garayoa M., San-Segundo L., Martín M., Mota I., Sanchez M.-L., Bárcena P., Aires-Mejia I. (2013). Detailed characterization of multiple myeloma circulating tumor cells shows unique phenotypic, cytogenetic, functional, and circadian distribution profile. Blood.

[21] Kraj M., Kopec-Szlezak J., Pogłód R., Kruk B. (2011). Flow cytometric immunophenotypic characteristics of 36 cases of plasma cell leukemia. Leuk. Res..

[22] Klimiene I., Radzevicius M., Matuzeviciene R., Sinkevic-Belliot K., Kucinskiene Z.A., Peceliunas V. (2021). Adhesion molecule immunophenotype of bone marrow multiple myeloma plasma cells impacts the presence of malignant circulating plasma cells in peripheral blood. Int. J. Lab. Hematol..

[23] Alunni-Fabbroni M., Sandri M.T. (2010). Circulating tumour cells in clinical practice: Methods of detection and possible characterization. Methods.

[24] Pantel K., Alix-Panabières C., Riethdorf S. (2009). Cancer micrometastases. Nat. Rev. Clin. Oncol..

[25] Paterlini-Brechot P., Benali N.L. (2007). Circulating tumor cells (CTC) detection: Clinical impact and future directions. Cancer Lett..

[26] Farace F., Massard C., Vimond N., Drusch F., Jacques N., Billiot F., Laplanche A., Chauchereau A., Lacroix L., Planchard D. (2011). A direct comparison of Cell Search and ISET for circulating tumour-cell detection in patients with metastatic carcinomas. Br. J. Cancer.

[27] Miller M.C., Doyle G.V., Terstappen L.W.M.M. (2010). Significance of circulating tumor cells detected by the CellSearch system in patients with metastatic breast colorectal and prostate cancer. J. Oncol..

[28] Cristofanilli M., Budd G.T., Ellis M.J., Stopeck A., Matera J., Miller M.C., Reuben J.M., Doyle G.V., Allard W.J., Terstappen L.W. (2004). Circulating tumor cells, disease progression, and survival in metastatic breast cancer. N. Engl. J. Med..

[29] Cohen S.J., Punt C.J.A., Iannotti N., Saidman B.H., Sabbath K.D., Gabrail N.Y., Picus J., Morse M., Mitchell E., Miller M.C. (2008). Relationship of circulating tumor cells to tumor response, progression-free survival, and overall survival in patients with metastatic colorectal cancer. J. Clin. Oncol..

[30] de Bono J.S., Scher H.I., Montgomery R.B., Parker C., Miller M.C., Tissing H., Doyle G.V., Terstappen L.W., Pienta K.J., Raghavan D. (2008). Circulating tumor cells predict survival benefit from treatment in metastatic castration-resistant prostate cancer. Clin. Cancer Res..

[31] Sun Y.-F., Yang X.-R., Zhou J., Qiu S.-J., Fan J., Xu Y. (2011). Circulating tumor cells: Advances in detection methods, biological issues, and clinical relevance. J. Cancer Res. Clin. Oncol..

[32] Williams A.L., Fitzgerald J.E., Ivich F., Sontag E.D., Niedre M. (2020). Short-Term Circulating Tumor Cell Dynamics in Mouse Xenograft Models and Implications for Liquid Biopsy. Front. Oncol..

[33] Ndacayisaba L.J., Rappard K.E., Shishido S.N., Ruiz Velasco C., Matsumoto N., Navarez R., Tang G., Lin P., Setayesh S.M., Naghdloo A. (2022). Enrichment-Free Single-Cell Detection and Morphogenomic Profiling of Myeloma Patient Samples to Delineate Circulating Rare Plasma Cell Clones. Curr. Oncol..

[34] Foulk B., Schaffer M., Gross S., Rao C., Smirnov D., Connelly M.C., Chaturvedi S., Reddy M., Brittingham G., Mata M. (2018). Enumeration and characterization of circulating multiple myeloma cells in patients with plasma cell disorders. Br. J. Hematol..

[35] Zhang L., Beasley S., Prigozhina N.L., Higgins R., Ikeda S., Lee F.Y., Marrinucci D., Jia S. (2016). Detection and characterization of circulating tumour cells in multiple myeloma. J. Circ. Biomark..

[36] Garcés J.J., Bretones G., Burgos L., Valdes-Mas R., Puig N., Cedena M.T., Alignani D., Rodriguez I., Puente D.Á., Álvarez M.G. (2020). Circulating tumor cells for comprehensive and multiregional non-invasive genetic characterization of multiple myeloma. Leukemia.

[37] Wang C., Xu Y., Li S., Zhou Y., Qian Q., Liu Y., Mi X. (2022). Designer tetrahedral DNA framework-based microfluidic technology for multivalent capture and release of circulating tumor cells. Mater. Today Bio.

[38] Xu X., Lin J., Guo Y., Wu X., Xu Y., Zhang D., Zhang X., Yujiao X., Wang J., Yao C. (2022). TiO_2_-based Surface-Enhanced Raman Scattering bio-probe for efficient circulating tumor cell detection on microfilter. Biosens. Bioelectron..

[39] Lohr J.G., Kim S., Gould J., Knoechel B., Drier Y., Cotton M.J., Gray D., Birrer N., Wong B., Ha G. (2016). Genetic interrogation of circulating multiple myeloma cells at single-cell resolution. Sci. Transl. Med..

[40] Mishima Y., Paiva B.D.L., Shi J., Park J., Manier S., Takagi S., Massoud M., Perilla-Glen A., Aljawai Y., Huynh D. (2017). The Mutational Landscape of Circulating Tumor Cells in Multiple Myeloma. Cell Rep..

[41] Zhan F., Huang Y., Colla S., Stewart J.P., Hanamura I., Gupta S., Epstein J., Yaccoby S., Sawyer J., Burington B. (2006). The molecular classification of multiple myeloma. Blood.

[42] Ledergor G., Weiner A., Zada M., Wang S.-Y., Cohen Y.C., Gatt M.E., Snir N., Magen H., Koren-Michowitz M., Herzog-Tzarfati K. (2018). Single cell dissection of plasma cell heterogeneity in symptomatic and asymptomatic myeloma. Nat. Med..

[43] Fokkema C., de Jong M.M.E., Tahri S., Kellermayer Z., den Hollander C., Vermeulen M., Papzian N., van Duin M., Wevers M.J.W., Sanders M.A. Abstract #1566: High Levels of Circulating Tumor Cells Are Associated with Increased Bone Marrow Proliferation in Newly Diagnosed Multiple Myeloma Patients. Proceedings of the 63rd ASH Annual Meeting & Exposition.

[44] Sanoja-Flores L., Flores-Montero J., Garcés J.J., Paiva B., Puig N., García-Mateo A., García-Sánchez O., Corral-Mateos A., Burgos L., Blanco E. (2018). Next generation flow for minimally invasive blood characterization of MGUS and multiple myeloma at diagnosis based on circulating tumor plasma cells (CTPC). Blood Cancer J..

[45] Bataille R., Jégo G., Robillard N., Barille-Nion S., Harousseau J.-L., Moreau P., Amiot M., Pellat-Deceunynck C. (2006). The phenotype of normal, reactive and malignant plasma cells. Identification of “many and multiple myelomas” and of new targets for myeloma therapy. Haematologica.

[46] Deceunynck C., Barille-Nion S., Jego G., Puthier D., Robillard N., Pineau D., Rapp M.-J., Harousseau J.-L., Amiot M., Bataille R. (1998). The absence of CD56 (NCAM) on malignant plasma cells is a hallmark of plasma cell leukemia and of a special subset of multiple myeloma. Leukemia.

[47] Lonial S., Jacobus S., Fonseca R., Weiss M., Kumar S., Orlowski R.Z., Kaufman J.L., Yacoub A.M., Buadi F.K., O’Brien T. (2020). Randomized trial of lenalidomide versus observation in smoldering multiple myeloma. J. Clin. Oncol..

[48] Garcés J.-J., Puig N., Termini R., Cedena M.-T., Moreno C., Pérez J.J., Alignani D., Sarvide S., Oriol A., González-García E. Abstract #76: Circulating Tumor Cells (CTCs) in Smoldering and Active Multiple Myeloma (MM): Mechanism of Egression, Clinical Significance and Therapeutic Endpoints. Proceedings of the 63rd ASH Annual Meeting & Exposition.

[49] Vasco-Mogorrón M.A., Campillo J.A., Periago A., Cabañas V., Berenguer M., García-Garay M.C., Gimeno L., Soto-Ramírez M.F., Martínez-Hernández M.D., Muro M. (2021). Blood-based risk stratification for pre-malignant and symptomatic plasma cell neoplasms to improve patient management. Am. J. Cancer Res..

[50] Garcés J.J., Cedena M.T., Puig N., Burgos L., Perez J.J., Cordon L., Flores-Montero J., Sanoja-Flores L., Calasanz M.J., Ortiol A. (2022). Circulating Tumor Cells for the Staging of Patients with Newly Diagnosed Transplant-Eligible Multiple Myeloma. J. Clin. Oncol..

[51] Manier S., Park J., Capelletti M., Bustoros M., Freeman S.S., Ha G., Rhoades J., Liu C.J., Huynh D., Reed S.C. (2018). Whole-exome sequencing of cell free DNA and circulating tumor cells in multiple myeloma. Nat. Commun..

[52] Jelinek T., Bezdekova R., Zatopkova M., Burgos L., Simicek M., Sevcikova T., Paiva B., Hajek R. (2017). Current applications of multiparameter fow cytometry in plasma cell disorders. Blood Cancer J..

[53] Huhn S., Weinhold N., Nickel J., Pritsch M., Hielscher T., Hummel M., Bertsch U., Huegle-Doerr B., Vogel M., Angermund R. (2017). Circulating tumor cells as a biomarker for response to therapy in multiple myeloma patients treated within the GMMG-MM5 trial. Bone Marrow Transplant..

[54] Li J., Wang N., Tesfaluul N., Gao X., Liu S., Yue B. (2019). Prognostic value of circulating plasma cells in patients with multiple myeloma: A meta-analysis. PLoS ONE.

[55] Nowakowski G.S., Witzig T.E., Dingli D., Tracz M.J., Gertz M.A., Lacy M.Q., Lust J.A., Dispenzieri A., Greipp P.R., Kyle R.A. (2005). Circulating plasma cells detected by flow cytometry as a predictor of survival in 302 patients with newly diagnosed multiple myeloma. Blood.

[56] Gonsalves W.I., Morice W.G., Rajkumar V., Gupta V., Timm M.M., Dispenzieri A., Buadi F.K., Lacy M.Q., Singh P.P., Kapoor P. (2014). Quantification of clonal circulating plasma cells in relapsed multiple myeloma. Br. J. Haematol..

[57] Bianchi G., Richardson P.G., Anderson K.C. (2015). Promising therapies in multiple myeloma. Blood.

[58] Flores-Montero J., Sanoja-Flores L., Paiva B., Puig N., García Sánchez O., Böttcher S., van der Velden V.H.J., Pérez-Morán J.J., Vidriales M.B., García-Sanz R. (2017). Next generation flow for highly sensitive and standardized detection of minimal residual disease in multiple myeloma. Leukemia.

[59] Giuliano M., Shaikh A., Lo H.C., Arpino G., De Placido S., Zhang X.H., Cristofanilli M., Schiff R., Trivedi M.V. (2018). Perspective on Circulating Tumor Cell Clusters: Why It Takes a Village to Metastasize. Cancer Res..

[60] Hou J.M., Krebs M.G., Lancashire L., Sloane R., Backen A., Swain R.K., Priest L.J., Greystoke A., Zhou C., Morris K. (2012). Clinical significance and molecular characteristics of circulating tumor cells and circulating tumor microemboli in patients with small-cell lung cancer. J. Clin. Oncol..

[61] Wang C., Mu Z., Chervoneva I., Austin L., Ye Z., Rossi G., Palazzo J.P., Sun C., Abu-Khalaf M., Myers R.E. (2017). Longitudinally collected CTCs and CTC-clusters and clinical outcomes of metastatic breast cancer. Breast Cancer Res. Treat..

[62] Macaraniag C., Luan Q., Zhou J., Papautsky I. (2022). Microfluidic techniques for isolation, formation, and characterization of circulating tumor cells and clusters. APL Bioeng..

[63] Snyder M.W., Kircher M., Hill A.J., Daza R.M., Shendure J. (2016). Cell-free DNA comprises an in vivo nucleosome footprint that informs its tissues-of-origin. Cell.

[64] Wan J.C.M., Massie C., Garcia-Corbacho J., Mouliere F., Brenton J.D., Caldas C., Pacey S., Baird R., Rosenfeld N. (2017). Liquid biopsies come of age: Towards implementation of circulating tumour DNA. Nat. Rev. Cancer.

[65] Kis O., Kaedbey R., Chow S., Danesh A., Dowar M., Li T., Li Z., Liu J., Mansour M., Masih-Khan E. (2017). Circulating tumour DNA sequence analysis as an alternative to multiple myeloma bone marrow aspirates. Nat Commun..

[66] Mithraprabhu S., Khong T., Ramachandran M., Chow A., Klarica D., Mai L., Walsh S., Broemeling D., Marziali A., Wiggin M. (2017). Circulating tumour DNA analysis demonstrates spatial mutational heterogeneity that coincides with disease relapse in myeloma. Leukemia.

[67] Bolli N., Avet-Loiseau H., Wedge D.C., Van Loo P., Alexandrov L.B., Martincorena I., Dawson K.J., Iorio F., Nik-Zainal S., Bignell G.R. (2014). Heterogeneity of genomic evolution and mutational profiles in multiple myeloma. Nat. Commun..

[68] Kumar S.K., Rajkumar S.V. (2018). The multiple myelomas—current concepts in cytogenetic classification and therapy. Nat. Rev. Clin. Oncol..

[69] Walker B.A., Mavrommatis K., Wardell C.P., Cody Ashby T., Bauer M., Davies F.E. (2018). Identification of novel mutational drivers reveals oncogene dependencies in multiple myeloma. Blood.

[70] Guo G., Raje N.S., Seifer C., Kloeber J., Isenhart R., Ha G., Yee A.J., O’Donnell E.K., Tai Y.T., Richardson P.G. (2018). Genomic discovery and clonal tracking in multiple myeloma by cell-free DNA sequencing. Leukemia.

[71] Gahan P.B., Swaminathan R. (2008). Circulating nucleic acids in plasma and serum. Recent developments. Ann. N. Y. Acad. Sci..

[72] Mandel P., Metais P. (1948). Les acides nucleiques du plasma sanguin chez l’homme. Seances Soc. Biol. Ses. Fil..

[73] Heitzer E., Auer M., Hoffmann E.M., Pichler M., Gasch C., Ulz P., Lax S., Waldispuehl-Geigl J., Mauermann O., Mohan S. (2013). Establishment of tumor-specific copy number alterations from plasma DNA of patients with cancer. Int. J. Cancer.

[74] Murtaza M., Dawson S.J., Tsui D.W., Gale D., Forshew T., Piskorz A.M., Parkinson C., Chin S.F., Kingsbury Z., Wong A.S. (2013). Non-invasive analysis of acquired resistance to cancer therapy by sequencing of plasma DNA. Nature.

[75] Cheng S.H., Jiang P., Sun K., Cheng Y.K., Chan K.C., Leung T.Y., Chiu R.W., Lo Y.M. (2015). Noninvasive prenatal testing by nanopore sequencing of maternal plasma DNA: Feasibility assessment. Clin. Chem..

[76] Lo Y.D., Zhang J., Leung T.N., Lau T.K., Chang A.M., Hjelm N.M. (1999). Rapid clearance of fetal DNA from maternal plasma. Am. J. Hum. Genet..

[77] Jahr S., Hentze H., Englisch S., Hardt D., Fackelmayer F.O., Hesch R.D., Knippers R. (2001). DNA fragments in the blood plasma of cancer patients: Quantitations and evidence for their origin from apoptotic and necrotic cells. Cancer Res..

[78] Dwivedi D.J., Toltl L.J., Swystun L.L., Pogue J., Liaw K.L., Weitz J.I., Cook D.J., Fox-Robichaud A.E., Liaw P.C. (2012). Prognostic utility and characterization of cell-free DNA in patients with severe sepsis. Crit. Care.

[79] Lim J.K., Kuss B., Talaulikar D. (2021). Role of cell-free DNA in haematological malignancies. Pathology.

[80] Gerber B., Manzoni M., Spina V., Bruscaggin A., Lionetti M., Fabris S., Barbieri M., Ciceri G., Pompa A., Forestieri G. (2018). Circulating tumor DNA as a liquid biopsy in plasma cell dyscrasias. Haematologica.

[81] Rustad E.H., Coward E., Skytøen E.R., Misund K., Holien T., Standal T., Børset M., Beisvag V., Myklebost O., Meza-Zepeda L.A. (2017). Monitoring multiple myeloma by quantification of recurrent mutations in serum. Haematologica.

[82] Weinhold N., Ashby C., Rasche L., Chavan S.S., Stein C., Stephens O.W., Tytarenko R., Bauer M.A., Meissner T., Deshpande S. (2016). Clonal selection and double-hit events involving tumor suppressor genes underlie relapse in myeloma. Blood.

[83] Hohaus S., Giachelia M., Massini G., Mansueto G., Vannata B., Bozzoli V., Criscuolo M., D’Alò F., Martini M., Larocca L.M. (2009). Cell-free circulating DNA in Hodgkin’s and non-Hodgkin’s lymphomas. Ann. Oncol..

[84] Schwarz A.K., Stanulla M., Cario G., Flohr T., Sutton R., Möricke A., Anker P., Stroun M., Welte K., Bartram C.R. (2009). Quantification of free total plasma DNA and minimal residual disease detection in the plasma of children with acute lymphoblastic leukemia. Ann. Hematol..

[85] Biancon G., Gimondi S., Vendramin A., Carniti C., Corradini P. (2018). Noninvasive Molecular Monitoring in Multiple Myeloma Patients Using Cell-Free Tumor DNA: A Pilot Study. J. Mol. Diagn. JMD.

[86] Manzoni M., Pompa A., Fabris S., Pelizzoni F., Ciceri G., Seia M., Ziccheddu B., Bolli N., Corradini P., Baldini L. (2020). Limits and Applications of Genomic Analysis of Circulating Tumor DNA as a Liquid Biopsy in Asymptomatic Forms of Multiple Myeloma. Hemasphere.

[87] Alidousty C., Brandes D., Heydt C., Wagener S., Wittersheim M., Schäfer S.C., Holz B., Merkelbach-Bruse S., Büttner R., Fassunke J. (2017). Comparison of Blood Collection Tubes from Three Different Manufacturers for the Collection of Cell-Free DNA for Liquid Biopsy Mutation Testing. J. Mol. Diagn. JMD.

[88] Mithraprabhu S., Spencer A. (2018). Analysis of Circulating Tumor DNA. Methods Mol. Biol. Clifton NJ.

[89] Long X., Xu Q., Lou Y., Li C., Gu J., Cai H., Wang D., Xu J., Li T., Zhou X. (2020). The utility of non-invasive liquid biopsy for mutational analysis and minimal residual disease assessment in extramedullary multiple myeloma. Br. J. Haematol..

[90] Rajkumar S.V., Landgren O., Mateos M.V. (2015). Smoldering multiple myeloma. Blood.

[91] Bolli N., Maura F., Minvielle S., Gloznik D., Szalat R., Fullam A., Martincorena I., Dawson K.J., Samur M.K., Zamora J. (2018). Genomic patterns of progression in smoldering multiple myeloma. Nat. Commun..

[92] Bustoros M., Sklavenitis-Pistofidis R., Park J., Redd R., Zhitomirsky B., Dunford A.J., Salem K., Tai Y.T., Anand S., Mouhieddine T.H. (2020). Genomic Profiling of Smoldering Multiple Myeloma Identifies Patients at a High Risk of Disease Progression. J. Clin. Oncol..

[93] Deshpande S., Tytarenko R.G., Wang Y., Boyle E.M., Ashby C., Schinke C.D., Thanendrarajan S., Zangari M., Zhan F., Davies F.E. (2021). Monitoring treatment response and disease progression in myeloma with circulating cell-free DNA. Eur. J. Haematol..

[94] Yasui H., Kobayashi M., Sato K., Kondoh K., Ishida T., Kaito Y., Tamura H., Handa H., Tsukune Y., Sasaki M. (2021). Circulating cell-free DNA in the peripheral blood plasma of patients is an informative biomarker for multiple myeloma relapse. Int. J. Clin. Oncol..

[95] Vrabel D., Sedlarikova L., Besse L., Rihova L., Bezdekova R., Almasi M., Kubaczkova V., Brožová L., Jarkovsky J., Plonkova H. (2020). Dynamics of tumor-specific cfDNA in response to therapy in multiple myeloma patients. Eur. J. Haematol..

[96] Mithraprabhu S., Hocking J., Ramachandran M., Choi K., Klarica D., Khong T., Reynolds J., Spencer A. (2019). DNA-Repair Gene Mutations Are Highly Prevalent in Circulating Tumour DNA from Multiple Myeloma Patients. Cancers.

[97] Waldschmidt J.M., Vijaykumar T., Knoechel B., Lohr J.G. (2020). Tracking myeloma tumor DNA in peripheral blood. Best Pract. Res. Clin. Haematol..

[98] Pugh T.J. (2018). Circulating Tumour DNA for Detecting Minimal Residual Disease in Multiple Myeloma. Semin. Hematol..

[99] Mazzotti C., Buisson L., Maheo S., Perrot A., Chretien M.-L., Leleu X., Hulin C., Manier S., Hébraud B., Roussel M. (2018). Myeloma MRD by Deep Sequencing from Circulating Tumor DNA Does Not Correlate with Results Obtained in the Bone Marrow. Blood Adv..

[100] Ntanasis-Stathopoulos I., Gavriatopoulou M., Terpos E., Fotiou D., Kastritis E., Dimopoulos M.A. (2020). Monitoring Plasma Cell Dyscrasias with Cell-Free DNA Analysis. Clin. Lymphoma Myeloma Leuk..

[101] Chawla S.S., Kumar S.K., Dispenzieri A., Greenberg A.J., Larson D.R., Kyle R.A., Lacy M.Q., Gertz M.A., Rajkumar S.V. (2015). Clinical Course and Prognosis of Non-Secretory Multiple Myeloma. Eur. J. Haematol.

[102] Dupuis M.M., Tuchman S.A. (2016). Non-secretory multiple myeloma: From biology to clinical management. OncoTargets Ther..

[103] Allegra A., Cicero N., Tonacci A., Musolino C., Gangemi S. (2022). Circular RNA as a Novel Biomarker for Diagnosis and Prognosis and Potential Therapeutic Targets in Multiple Myeloma. Cancers.

[104] Allegra A., Ettari R., Innao V., Bitto A. (2021). Potential Role of microRNAs in inducing Drug Resistance in Patients with Multiple Myeloma. Cells.

[105] Avenoso A., Campo S., Scuruchi M., Mania M., Innao V., D’Ascola A., Mandraffino G., Allegra A.G., Musolino C., Allegra A. (2021). Quantitative polymerase Chain reaction profiling of microRNAs in peripheral lymph-monocytes from MGUS subjects. Pathol. Res. Pract..

[106] Musolino C., Oteri G., Allegra A., Mania M., D’Ascola A., Avenoso A., Innao V., Allegra A.G., Campo S. (2018). Altered microRNA expression profile in the peripheral lymphoid compartment of multiple myeloma patients with bisphosphonate-induced osteonecrosis of the jaw. Ann. Hematol..

[107] Ye J., Xu M., Tian X., Cai S., Zeng S. (2019). Research advances in the detection of miRNA. J. Pharm. Anal..

[108] Kshirsagar P., Seshacharyulu P., Muniyan S., Rachagani S., Smith L.M., Thompson C., Shah A., Mallya K., Kumar S., Jain M. (2022). DNA-gold nanoprobe-based integrated biosensing technology for non-invasive liquid biopsy of serum miRNA: A new frontier in prostate cancer diagnosis. Nanomedicine.

[109] Chen M., Mithraprabhu S., Ramachandran M., Choi K., Khong T., Spencer A. (2019). Utility of Circulating Cell-Free RNA Analysis for the Characterization of Global Transcriptome Profiles of Multiple Myeloma Patients. Cancers.

[110] Wang J.J., Liu Y., Ding Z., Zhang L., Han C., Yan C., Amador E., Yuan L., Wu Y., Song C. (2022). The exploration of quantum dot-molecular beacon based MoS_2_ fluorescence probing for myeloma-related Mirnas detection. Bioact. Mater..

[111] Xu R., Rai A., Chen M., Suwakulsiri W., Greening D.W., Simpson R.J. (2018). Extracellular vesicles in cancer—Implications for future improvements in cancer care. Nat. Rev. Clin. Oncol..

[112] Manier S., Liu C.J., Avet-Loiseau H., Park J., Shi J., Campigotto F., Salem K.Z., Huynh D., Glavey S.V., Rivotto B. (2017). Prognostic role of circulating exosomal miRNAs in multiple myeloma. Blood.

[113] Sedlarikova L., Bollova B., Radova L., Brozova L., Jarkovsky J., Almasi M., Penka M., Kuglik P., Sandecka V., Stork M. (2018). Circulating exosomal long noncoding RNA PRINS-First findings in monoclonal gammopathies. Hematol. Oncol..

[114] Allegra A., Petrarca C., Di Gioacchino M., Casciaro M., Musolino C., Gangemi S. (2022). Exosome-Mediated Therapeutic Strategies for Management of Solid and Hematological Malignancies. Cells.

[115] Allegra A., Di Gioacchino M., Tonacci A., Petrarca C., Musolino C., Gangemi S. (2021). Multiple Myeloma Cell-Derived Exosomes: Implications on Tumorigenesis, Diagnosis, Prognosis and Therapeutic Strategies. Cells.

[116] Hoshino A., Costa-Silva B., Shen T.L., Rodrigues G., Hashimoto A., Tesic Mark M., Molina H., Kohsaka S., Di Giannatale A., Ceder S. (2015). Tumour Exosome Integrins Determine Organotropic Metastasis. Nature.

[117] Peinado H., Alečković M., Lavotshkin S., Matei I., Costa-Silva B., Moreno-Bueno G., Hergueta-Redondo M., Williams C., García-Santos G., Ghajar C.M. (2012). Melanoma Exosomes Educate Bone Marrow Progenitor Cells Toward a Pro-Metastatic Phenotype Through MET. Nat. Med..

[118] Tutrone R., Donovan M.J., Torkler P., Tadigotla V., McLain T., Noerholm M., Skog J., McKiernan J. (2020). Clinical Utility of the Exosome Based ExoDx Prostate (IntelliScore) EPI Test in Men Presenting for Initial Biopsy with a PSA 2–10 Ng/Ml. Prostate Cancer Prostatic Dis..

[119] Reale A., Carmichael I., Xu R., Mithraprabhu S., Khong T., Chen M., Fang H., Savvidou I., Ramachandran M., Bingham N. (2021). Human Myeloma Cell- and Plasma-Derived Extracellular Vesicles Contribute to Functional Regulation of Stromal Cells. Proteomics.

[120] Reale A., Khong T., Mithraprabhu S., Spencer A. (2021). Translational Potential of RNA Derived From Extracellular Vesicles in Multiple Myeloma. Front. Oncol..

[121] Fan W., Han P., Feng Q., Sun Y., Ren W., Lawson T., Liu C. (2022). Nucleic Acid Substrate-Independent DNA Polymerization on the Exosome Membrane: A Mechanism Study and Application in Exosome Analysis. Anal. Chem..

[122] Fattahi Z., Khosroushahi A.Y., Hasanzadeh M. (2020). Recent progress on developing of plasmon biosensing of tumor biomarkers: Efficient method towards early stage recognition of cancer. Biomed. Pharmacother..

[123] Farhana F.Z., Umer M., Saeed A., Pannu A.S., Shahbazi M., Jabur A., Nam H.J., Ostrikov K., Sonar P., Firoz S.H. (2021). Isolation and detection of exosomes using Fe2O3 nanoparticles. ACS Appl. Nano Mater..

[124] Li S., Ma Q. (2022). Electrochemical nano-sensing interface for exosomes analysis and cancer diagnosis. Biosens. Bioelectron..

[125] Caivano A., La Rocca F., Simeon V., Girasole M., Dinarelli S., Laurenzana I., De Stradis A., De Luca L., Trino S., Traficante A. (2017). MicroRNA-155 in Serum-Derived Extracellular Vesicles as a Potential Biomarker for Hematologic Malignancies–A Short Report. Cell Oncol..

[126] Kubiczkova L., Kryukov F., Slaby O., Dementyeva E., Jarkovsky J., Nekvindova J., Radova L., Greslikova H., Kuglik P., Vetesnikova E. (2014). Circulating Serum microRNAs as Novel Diagnostic and Prognostic Biomarkers for Multiple Myeloma and Monoclonal Gammopathy of Undetermined Significance. Haematologica.

[127] Zhang Z.Y., Li Y.C., Geng C.Y., Wang H.J., Chen W.M. (2019). Potential Relationship Between Clinical Significance and Serum Exosomal miRNAs in Patients with Multiple Myeloma. BioMed Res. Int..

[128] Gong J., Jaiswal R., Mathys J.M., Combes V., Grau G.E., Bebawy M. (2012). Microparticles and their emerging role in cancer multidrug resistance. Cancer Treat. Rev..

[129] Innao V., Rizzo V., Allegra A.G., Musolino C., Allegra A. (2021). Promising Anti-Mitochondrial Agents for Overcoming Acquired Drug Resistance in Multiple Myeloma. Cells.

[130] Allegra A., Casciaro M., Barone P., Musolino C., Gangemi S. (2022). Epigenetic Crosstalk between Malignant Plasma Cells and the Tumour Microenvironment in Multiple Myeloma. Cancers.

[131] Rajeev Krishnan S., De Rubis G., Suen H., Joshua D., Lam Kwan Y., Bebawy M. (2020). A liquid biopsy to detect multidrug resistance and disease burden in multiple myeloma. Blood Cancer J..

[132] Leslie M. (2010). Cell Biology. Beyond Clotting: The Powers of Platelets. Science.

[133] Nilsson R.J.A., Balaj L., Hulleman E., van Rijn S., Pegtel D.M., Walraven M., Widmark A., Gerritsen W.R., Verheul H.M., Vandertop W.P. (2011). Blood Platelets Contain Tumor-Derived RNA Biomarkers. Blood.

[134] Meng Y., Sun J., Zheng Y., Zhang G., Yu T., Piao H. (2021). Platelets: The Emerging Clinical Diagnostics and Therapy Selection of Cancer Liquid Biopsies. Oncotargets Ther..

[135] Shah U.J., Alsulimani A., Ahmad F., Mathkor D.M., Alsaieedi A., Harakeh S., Nasiruddin M., Haque S. (2022). Bioplatforms in liquid biopsy: Advances in the techniques for isolation, characterization and clinical applications. Biotechnol. Genet. Eng. Rev..

[136] Wang L., Wang X., Guo E., Mao X., Miao S. (2022). Emerging roles of platelets in cancer biology and their potential as therapeutic targets. Front. Oncol..

[137] Gao P., Xiao Z.P., Fu K., Han M. (2017). Clinical significance of mean platelet volume determination in multiple myeloma. Zhongguo Shi Yan Xue Ye Xue Za Zhi..

[138] Zhuang Q., Xiang L., Xu H., Fang F., Xing C., Liang B., Yu K., Feng J. (2016). The independent association of mean platelet volume with overall survival in multiple myeloma. Oncotarget.

[139] Flach J., Shumilov E., Joncourt R., Porret N., Novak U., Pabst T., Bacher U. (2020). Current concepts and future directions for hemato-oncologic diagnostics. Crit. Rev. Oncol. Hematol..

[140] Cavo M., Terpos E., Nanni C., Moreau P., Lentzsch S., Zweegman S., Hillengass J., Engelhardt M., Usmani S.Z., Vesole D.H. (2017). Role of 18F-FDG PET/CT in the diagnosis and management of multiple myeloma and other plasma cell disorders: A consensus statement by the International Myeloma Working Group. Lancet Oncol..

[141] López-Anglada L., Gutiérrez N.C., García J.L., Mateos M.V., Flores T., San Miguel J.F. (2010). P53 deletion may drive the clinical evolution and treatment response in multiple myeloma. Eur. J. Haematol..

